# A Review of Wearable Sensor Systems for Monitoring Body Movements of Neonates

**DOI:** 10.3390/s16122134

**Published:** 2016-12-14

**Authors:** Hongyu Chen, Mengru Xue, Zhenning Mei, Sidarto Bambang Oetomo, Wei Chen

**Affiliations:** 1 Center for Intelligent Medical Electronics, Department of Electronic Engineering, School of Information Science and Technology, Fudan University, Shanghai 200433, China; chenhongyudesign@outlook.com (H.C.); 16210720037@fudan.edu.cn (Z.M.); 2 Department of Industrial Design, Eindhoven University of Technology, Eindhoven 5600 MB, The Netherlands; m.xue@tue.nl (M.X.); S.BambangOetomo@mmc.nl (S.B.O.); 3 Department of Neonatology, Máxima Medical Center, Veldhoven 5500 MB, The Netherlands; 4 Shanghai Key Laboratory of Medical Imaging Computing and Computer Assisted Intervention, Shanghai 200000, China

**Keywords:** infant, wearable sensor, movement monitoring, neonate, seizure

## Abstract

Characteristics of physical movements are indicative of infants’ neuro-motor development and brain dysfunction. For instance, infant seizure, a clinical signal of brain dysfunction, could be identified and predicted by monitoring its physical movements. With the advance of wearable sensor technology, including the miniaturization of sensors, and the increasing broad application of micro- and nanotechnology, and smart fabrics in wearable sensor systems, it is now possible to collect, store, and process multimodal signal data of infant movements in a more efficient, more comfortable, and non-intrusive way. This review aims to depict the state-of-the-art of wearable sensor systems for infant movement monitoring. We also discuss its clinical significance and the aspect of system design.

## 1. Introduction

Neonatology is a subspecialty of pediatrics that cares for newborn infants. The most important task for a neonatologist is to give appropriate treatment and to provide optimal nursing conditions [1]. Normal neurodevelopment is highly dependent on the well-being of the brain. Complications of preterm birth and diseases that occur in the perinatal period can affect the brain [2]. Therefore, it is important to assess brain function of the newborn that is at risk for neuro-developmental impairments. The methods that are used presently for neonatal brain monitoring include ultra-sonography [3], magnetic resonance imaging [4], electroencephalography [5], and near infrared spectroscopy [6]. These modalities are applied in patients suffering from diseases that affect the brain, like intracranial hemorrhage, meningitis, and hypoxic ischemic encephalopathy [7]. The observation of clinical signs of brain dysfunction, like seizures, can only be made when a caretaker is in the presence of the patient. Seizures usually manifests as abnormal movements of the limbs and eyes. The occurrence and type of seizure can be appreciated by observing the nature, speed, and amplitude of the movements [8,9]. It is obvious that seizures may be neglected and undiagnosed when infants are not closely surveyed. Presently, newborn infants that are at high risk for seizures are usually admitted in a neonatal intensive care unit, where monitoring of the vital signs and an electroencephalogram (EEG) are applied. The multitude of sensors and cables may cause discomfort to neonates, increase the complexity of clinical work and limit the physical contact between the parents and their baby. Therefore we explore technologies that can monitor body movements and identify abnormal movements without unnecessarily sacrificing the degree of comfort.

Another clinical scenario which requires the monitoring of movements is the assessment of general movements. This is a method which describes the motor behavior of developing babies in a qualitative way [10]. However, this method is developed mainly as a research tool and is applied clinically only in dedicated centers. One major limitation of this method is the high inter-observer variability, which is due to the qualitative nature. The methods that are presently used for qualitative analysis of infants’ motor behaviors include stereo photogrammetric movement analysis and a gaze-tracking-based approach. Studies on reaching/grasping tasks in children, for example, have been conducted by means of camera-based motion capture systems with passive markers. This approach requires a highly structured environment (i.e., laboratories), which is highly dependent on the doctors’ experiences [11].

In recent years, with the soaring development of wearables and sensors, including accelerometers, gyroscopes, smart fabrics, actuators, as well as the progress in wireless communication networks, power supplies, and data-capture technology for signal processing and decision support [12], the monitoring of movements has become a hot area [13]. One recent development is the adoption of wearable motion-sensing technology for studying infant movements. Based on the utilization of miniaturized motion sensors, long-term monitoring of daily activity and assessment of motor functioning in infants without disturbance becomes more practical than ever before. These methods are highly relevant for the research on the motor pattern of infants, but the clinical effect still has to be validated.

There are related reviews about wearable sensor systems for motion sensing technology and infants. Marcroft et al. presented the state-of-art about movement recognition technology for assessing spontaneous general movements in high-risk infants without paying attention to the influence from the form aspect. Both wearables and other forms are mentioned averagely [14]. Zhu et al. provided a comprehensive overview about wearable sensor systems aiming to measure different kinds of physiological signals of infants. They launched their review in a bottom-up approach, from sensors to system architectures [13].

The primary aim of this paper is to survey the state-of-the-art technology on infant movement monitoring based on wearable sensor systems. The authors searched for literature published after 2010 using a systematic review method. Since then the related work started to emerge. In Section 2, we introduce the search method. In Section 3 the sketch of search result is given. Section 4 discusses the state-of-the-art wearable sensor technology together with its clinical relevance and the topic of system design. In Section 5, the summary and the future prospects are elaborated.

## 2. Methods

### 2.1. Literature Research Strategy

The primary purpose of this paper is to review the wearable sensor system for monitoring body movements in infants. A search on following publication database was conducted: PubMed (MEDLINE since 1960), IEEE Xplore, SpringerLink and Science Direct, issued in February 2016. Relevant articles in the past seven years (2010–2016) were collected. To seek out related articles, we target the following three aspects: Infant, movement monitoring, and wearable sensor systems. Generic search terms (according to the thesaurus of each individual database) were used for the identification of relevant studies. Due to the different format of each database, we used slightly different expressions of our search strategy for each database. Table 1 displays the search strategy for the PubMed database. The search strategies for other three databases resemble to this. Only papers in English were included in the review process.

### 2.2. Study Selection Process

The selection procedure consists of two steps. The first selection was performed based on the title, the abstract and the identifying of exclusion criteria: 

Exclusion criteria were:

- No infant target population;
- No wearable sensor technology;
- No “movement” or “monitoring” in the research;
- Reviews;
- Books of conference proceeding;
- Language other than English.

We applied such exclusion criteria for obtaining desired relevant results which were well confined within the scope we are interested in.

The second step was based on full-text scan of the paper. Papers were included in the full-text review when they satisfied all of the following inclusion criteria.

Inclusion criteria were:

- All studies with infants as subjects.
- Technology: wearable motion-sensing technology.
- Related “body movement” or “moving” or “motor pattern” had to be reported.

During the second step, other two persons (Wei Chen, Zhenning Mei) decided whether the article should be included in the review.

### 2.3. Defination of Keywords in This Review

● Infant

The term infant in this review is defined as young children aged from newborn to two years old.

● Movement

In this review we focus on infant’s movement monitoring. Thus, mobility characteristics include position, motion, posture, activity, motor behavior, moving, eye deviation, fixed open stare, blinking, apnea, cycling, boxing, stepping, swimming movement of limbs, mouthing, chewing, and lip smacking [8].

● Wearable motion-sensing technology

“Wearable motion-sensing technology” represents the recording of a movement by means of small wearable or portable personal technologies as part of a network of physical objects or “things” embedded with electronics, software, sensors and connectivity. This technology enables objects to exchange data with a manufacturer, operator and/or other connected devices automatically and adaptively [15].

### 2.4. Screening Process

**Figure 1:**
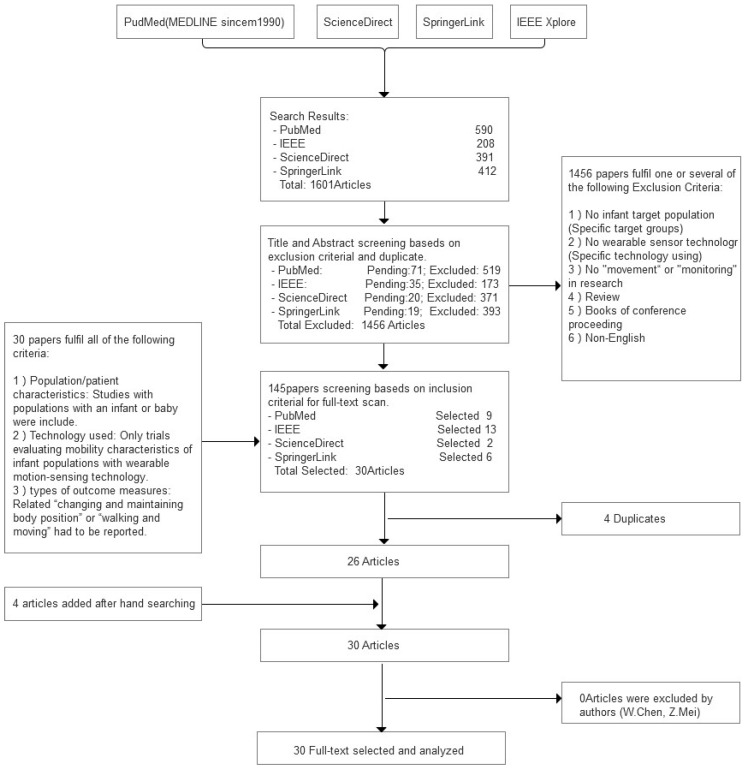
The procedure for study selection with databases used for the literature research.

Figure 1 shows the selection procedure and results. There are 1165 articles selected by our search strategy. After reading the titles and abstracts, we identified 145 papers based on the exclusion criteria. Two articles were excluded because they were written in languages other than English. After application of the inclusion criteria, the number was reduced to 30, and after removing four duplicates, 26 papers remained for consideration. We manually searched the references for these 26 articles. Four articles were added after hand searching of references. Finally 30 papers were taken into account in this review after two authors (Wei Chen, Zhenning Mei) carried out the decision on the 30 selected papers. This resulted in 30 articles that were considered in this review.

## 3. Results

With the development of sensor technology and wireless communication technology, the research on movement monitoring with wearable sensor systems for infants has made a lot of progress. Wearable sensor systems are becoming smaller, more intelligent, and many of them are commercially available [16]. These sensor systems have been embedded in more and more diversified products, such as shoes, buttons, belts, clothes etc., for the purpose of movement monitoring. A typical infant movement monitoring system with wearable sensors is commonly composed of sensors, power supplies, wireless communication modules and links, control and processing units, interfaces for the users (e.g., for parents or doctors), software, and algorithms for signal processing, feature extraction, and decision-making. Table 2 summarizes recent work from 2010–2016.

We have seen a number of fruitful results approaching the topic of wearable sensor systems for infant movement monitoring. They vary in the aspects of sensor type, placement, exterior structure, and evaluation for the purposes ranged from movement assessment to artifacts reduction.

Among the 30 selected articles, a total of five types of sensors, including accelerometers, inertial measurement units (IMUs), magneto-inertial, pressure sensors, and flexible sensors are discussed. However, only 19 of them are described in the form of wearable systems, such as gadgets, bands, and jackets. Ten studies use the form of banding, including cloth bands, belt, bracelets, and so on. Two studies applied gadgets and another two implemented mats. The form of shoes and socks was also used by two researchers. However, only three use the form of jackets and suits. Most studies place the sensors on the hands or feet. Some also combine the sensors with other parts of the body, such as the forehead. Additionally, three studies put the sensors on the abdomen or the chest. Six passages illustrate the weight of the sensor and show that lighter sensors exert less influence upon the research results.

Regarding the purpose of sensor systems, 12 articles are about infant motor pattern assessment, three articles are used to predict cerebral palsy, and 13 are applied for baby safety. None of the 30 articles mentioned seizures. 

Thirteen articles demonstrated the technical tests on system feasibility. Fourteen research works carried out clinical tests or pre-clinical tests. Most of the tests were not large trials because the movement sample capacity was below 25. Only two tests had sample sizes larger than 25; one was 33, and the other was 75.

## 4. Discussion

### 4.1. Wearable Sensor Technologies for Infant Movement Monitoring

Various kinds of sensors were used for the monitoring of movements in infants, including accelerometers, gyroscopes, and magnetosensors, etc. Different types of sensors have advantages and disadvantages. Therefore, it is crucial to choose an appropriate sensor that meets the specific requirements for monitoring the movements of infants. In this section, we discuss the wearable sensors used in infant movement monitoring. 

Microelectromechanical systems (MEMS) is a process technology used to create tiny integrated devices or systems. It is the integration of mechanical elements, actuators, and electronics on a common silicon substrate through the utilization of microfabrication technology. One of the applications of MEMS technology is the MEMS-based inertial sensor.

Inertial sensors, also known as inertial measurement units (IMUs), consisting of accelerometers, gyroscopes, and/or magnetometers, are some of the most important types of silicon-based sensors. They gather movement information by measuring acceleration, angular rate, and the magnetic field vector (some of them) in the three axes of their own three-dimensional local coordinate system, respectively [45,46].

In the area of MEMS-based inertial sensors, major developments have been achieved recently, including their satisfactory sizes, low costs, and low power consumption, which have made inertial sensors prevalent in physical activity monitoring [47]. 

Recently, inexpensive on-chip inertial sensors, including gyroscopes and accelerometers, have gradually found practical applications in baby motion analysis.

Fabrizio Taffoni et al. [11] proposed a magneto-inertial platform, composed of three sensors: two wired magneto-inertial sensors that can be worn by infants on their wrists to evaluate upper limb movements. Hirotaka Gima et al. [22] proposed a low-cost system based on accelerometers to evaluate a newborn’s movement.

In some studies [38,41,42], flexible sensors are also used to monitor infant movement, such as bend sensors, which are usually used to acquire joint angle data, the Jew motion sensor is used to capture the action information of mouth mastication. Flexible sensors can acquire knowledge about the posture of static objects, which is beyond the ability of IMUs. Moreover, flexible sensors are thin and light, easy to be adopted in wearables. Thus, the flexible sensor is also suitable for the monitoring of infant movement, especially in quasi-static scenarios or for interest in an object’s posture. On the other hand, pressure sensors are also used. They are often embedded in a non-wearable system to collect activity-related data, without affecting the baby’s normal activities, such as mats [17,23,34].

For infant movement monitoring, various types of sensors have been used, as is shown in Table 3. The data in Table 3 are from literature search results from 2010–2016. For some flexible sensors working in the form of pressure sensing, we take them as flexible sensors to emphasize their flexibility, which is related to system design, while the sensing principal is not what we are interested in. As illustrated in the table, a majority of studies use accelerometers as their primary sensors. Fifty percent of all of the research papers have mentioned the use of accelerometers for this purpose. Motor characterization of infant general movement with inertial sensors has already given rise to several scientific contributions. For instance, Rihar et al. provided a study about the motor characterization of infant trunk posture and arm movement assessment performed with a multi-sensor measurement system [17].

In conclusion, with the development of the background technique, inertial sensors (IMU, accelerometer, magneto-inertial) are about to take the spotlight in the motion sensing area. Due to their low-cost, portable size, and high performance, increased numbers of researchers are considering these sensors as good choices for movement monitoring in infants.

### 4.2. Clinical Relevance of Movement Monitoring in Infant with Wearable Sensor System

Infant movement contains valuable information with clinical significance. However, most of the information never becomes available and practical due to the lack of efficient methods to extract it. Although some devices that are using MEMS technology were commercially successful, especially for those applications that require moderate accuracy (e.g., [48]), these kinds of sensor systems for medical applications have not really entered the market yet. Wearable sensor systems for monitoring movements in infants can also be used in ambulatory monitoring to continuously measure long-term movements of subjects in an independent environment. Related signal analysis algorithms recognize postures and classify movements into different categories which are related to infants’ functional status from recorded longitudinal activity raw data.

The good news is that some recently-developed, complete, multi-sensor systems are primarily designed for tackling this problem, which can also be exploited to a greater potential for its medical use in infants [17]. Among them, inertial sensors are being used in a wide range of medical applications for movement assessment, seizure alarms, and other scenarios where early intervention is needed. 

In this section, the indications for infant movement monitoring with wearable sensor systems are reviewed separately based on some applications. Table 4 shows the different purposes of infant movement monitoring from 2010–2016.

#### 4.2.1. Infant Movement and Motor Pattern

Patterns of motions in early life can predict impairments in neuro-motor development. It is a crucial step in identifying neuro-motor impairments and perfecting therapeutic approaches to evaluate infants’ physical activity patterns [49,50,51]. Qualitative and quantitative methods have been applied to studies in spontaneous movements (SMs) in infants. According to the observation of spontaneous motor activity, Prechtl and coworkers developed, to evaluate the motor integrity of infants, a qualitative method [10] which, as they have described, can assess the quality of motion patterns in infants. They also brought up the term ‘general movements’ (GMs) which illustrates whole-body movements characterized by changing speed, amplitude, and sequence [10,52,53].

Newborns and young infants accept GM observations, up to the post term of 20 weeks of age. As shown in Table 5, GMs will show specific characteristics corresponding to their ages, respectively [54].

In the last decades general movement assessment (GMA) became a new type of newborn motor assessment, which can be used as a diagnostic tool for the evaluation of function of young nervous systems. The so-called GMA has been described by Einspieler and Bos [10,55]. Although seemingly effective, it does not imply that GMA is easily applicable in a clinical setting. This is probably because of its subjectivity. 

In summary, current techniques for infant movement monitoring mostly rely on expert observer scoring, which is limited by skill, fatigue, and inter-rater variability. 

Gravem et al. present a clinical tool, using a mini-accelerometer, assessing patterns of movement in preterm infants [26]. Data quality and reliability are provided with wearable sensors, such as accelerometers, to assess the characteristics of movement disorders [21]. 

#### 4.2.2. Assessment Function of Cerebral Nervous System

Cerebral palsy (CP) is due to abnormal development or damage to the parts of the brain that control movement, balance, and posture [56]. It can develop after severe injuries in the perinatal period, including brain hemorrhage, lack of oxygen, and infection. Infancy or early childhood is the period when signs of CP become visible and takes a toll on body motion and coordination performance [57]. CP is a clinical diagnosis made by doctors’ observation of spontaneous movements and neurological examinations. There is empirical evidence that markedly abnormal movements reflect the existence of serious brain dysfunction [55]. So far, serial assessments of GMs can help to predict CP. In this respect the absence of fidgety movements (FMs) and the observation of cramped–synchronized general movements (CSGM) are associated with the development of CP. CSGMs look rigid, characterized by all limb and trunk muscles, with almost concurrent contracting and relaxing [19]. A high rate (93%) of CP has been shown in the follow-up of preterm babies that persisted to display CSGMs. The appearance of CSGMs indicates the existence of CP [58]. 

In cerebral palsy, studies aim at distinguishing pathological and normal movements whereas, in ecological behavioral studies, the goal is to classify different types of ecological behaviors using inertial sensors. In these cases, it is important to acquire the accurate knowledge of the angular motion of arms and legs. Angular velocities are typically measured by rate gyroscopes, which are particularly susceptible to drift. Accelerometers, on the other hand, cannot be used alone since they do not provide sufficient information. Therefore, to obtain better results, accelerometers are typically used with gyroscopes to construct an IMU [59]. When appropriate combinations of these sensors are deployed, it satisfies the requirements of data fusion.

Mohan Singh et al. reported a system that leverages accelerometers to detect CSGM in premature babies. Fan et al. have developed a Markov model-based technique that recognizes gestures from accelerometers. They show that by treating instantaneous machine learning classification values as observations and, explicitly, modeling duration, improved performance on the recognition of CSGM is obtained [24].

Seizures are a clinical sign of a functional disturbance of the cerebral nervous system. Seizure occurs on people more commonly during their infancy than any other time before they become children [60]. Seizure detection in babies is important. Seizures can be recognized as repetitive movements of an arm, hand, leg, and eye, in general, with an alternating slow and fast contraction of muscles in opposite directions. Apart from these manifestations, newborn infants can feature subtle convulsions, including eye deviation, fixed open stare, blinking, apnea, cycling, boxing, stepping, swimming movements of the limbs, mouthing, chewing, and lip smacking, or tonic and clonic convulsions, like stiffening, decerebrate posturing, and unifocal/multifocal repetitive jerking, or myoclonic seizures, more specifically [8,9]. These subtle movements are more difficult to interpret as seizures because most seizures occur unpredictably with potential outcomes of trauma and the risk of death [61]. In some cases, the symptoms of seizures may be overlooked when infants are unmonitored, especially when they are asleep, which increases the difficulty for diagnosis. On such occasions, a shortage of immediate medical assistance can cause even higher risks of mortality [62]. The development of a system that can detect and record the seizure and furthermore trigger an alarm on occasions when there is a high possibility of seizures is critical for reducing the risks caused by it. With an adequate seizure detection system, hospital staff can be warned and, thus, they will know precisely how often, when, and under what circumstances the seizure will occur in a particular patient.

The literature search in the area shows that most of the sensors presently applied are based on video recording or EEG, together with inertial sensors, to detect epileptic episodes in infants. To detect nocturnal hypermotor seizure in pediatric patients, Van de Vel invented a system that relies on an accelerometer (ACM) attached to the extremities [63]. Accelerometers have been used by detecting seizures in adults and children [64]. However, it has not been applied to infants.

#### 4.2.3. Other Clinical Relevance

Movement monitoring has great potential in early intervention, which aims at improving cognitive and motor outcomes of preterm infants [65]. 

It will encourage kicking, improving joint coordination, and gait development. By incorporating knowledge of infant learning into the design of a therapy device, spontaneous kicking can be encouraged in developmentally-delayed infants by providing an external stimulus in response to kicking [42]. 

Emily et al. present a soft spandex suit with sensors on the hips and knees, a control box, an external stimulus, and a graphical user interface (GUI). The sensor suit is a soft, flexible, spandex one-piece suit that can be comfortably worn by the infant with zippers on the legs and arms. Thin pockets on the knees and hips hold the sensors in place and prevent contact with the infant’s skin. A microcontroller was programmed to read the signal from the joint angle sensors, send the sensor data to the computer, and output the appropriate response to control the mobile. The mobile is attached to a DC motor that spins when infant kicking is detected [42].

Early intervention can also be used as a precautionary treatment for infants at risk even before an official diagnosis is possible [42]. For example, cerebral palsy is difficult to diagnose before the age of two, many infants do not begin receiving treatment until after gait and movement abnormalities have caused secondary health problems [66]. 

Other clinical applications of neonatal movement monitoring also include motor skill studies for infants at risk for autism spectrum disorders [11], safe sleep [16,23,33,61,62,63,67,68,69,70,71,72,73,74], and fall protection [29]. The knowledge and experience of wearable sensor systems for the aforementioned applications can be transferred to the exploitation of a neonatal seizure detection system.

#### 4.2.4. Motion Artifacts Reduction

All of the systems and methods mentioned above cannot become practical without high-grade signals. In this paper, the motion signal is used with reference to ECG signal. The author uses the motion signal to find the diversity of signal quality, which can be indicative of artifacts. The correlation between signal quality and context information are also discussed [36].

Furthermore, multi-modal physiological signals can be combined with movement signals through data fusion techniques to improve the performance of related decision-making systems [75].

### 4.3. System Design

#### 4.3.1. Tendency of Utilization of Wearable Sensors

Among the data demonstrated above, researchers have chosen various forms of sensors to monitor infant movements. For instance, of the 30 articles, 20 papers use accelerometer sensors to monitor babies, while three of them use pressure sensors.

**Figure 2:**
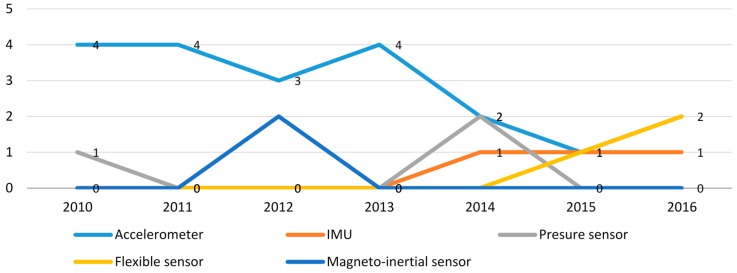
Trends of sensor technology usage in baby-related movement monitoring research.

Figure 2 illustrates the baby-related movement monitoring research from 2010–2016; the usage rate of the acceleration sensor is decreasing gradually, while that of the IMU is obviously increasing. That is because IMU measurements provide data with more degrees of freedom for movement monitoring.

#### 4.3.2. Exterior Structure

On the other hand, the exterior structure does make a difference on comfortability and user experience, which is an aspect to be considered in the design process. Table 4 demonstrates various wearable forms for monitoring infant movement, including gadgets, soft belts, bracelets, cloth bands, and jackets. Lee proposed a band that was built of medical-grade silicone integrated with an accelerometer to track the baby’s position and motion, a contactless sensor to gauge the baby’s temperature and an optical heart-rate sensor to monitor the baby’s pulse [16]. In [16], the band is wrapped around the ankle in a comfortable way to the infant, and the sensors are wrapped in medical-grade silicone and easy-to-remove non-wearable components. The accelerometer sensor is directly bound with the infant’s ankle and placed above the foot so that the position of the lower leg can be monitored the best and, in this way, it is accurate and convenient to obtain the baby’s body position and its movement and, thus, if the baby falls or rolls over, it can give a warning signal to the parents. Another novel and distinctive part of this band is that a base station doubles as a wireless charger, using magnetic resonance to provide enough power to run the band for one to two days at a time. Moreover, the data were sent wirelessly instead of using wired transmission and that improves the comfortability significantly. Rihar et al. [17], Gravem et al. [26], Hayes et al. [30], and Fan et al. [24] use a similar form of the system to monitor infant movement. 

#### 4.3.3. Design Criterion

For more specific tasking scenarios, like general movement monitoring in infants, the appearance design needs to satisfy the aesthetic requirements, the system needs to be small and light enough, and it should not restrict the infants’ movements and other actions. Additionall, risks of radiation and other kinds of health hazards should be eliminated [76]. 

In Bouwstra’s research, she put forward the following requirements for wearable sensor system design for neonatal monitoring which can be used for reference in further investigations [77]:

- Be able to achieve continuous monitoring when the infant is inside an incubator or during Kangaroo mother care.
- Be non-intrusive and avoid disturbance of infants and avoid causes of stress or stimuli.
- Be safe to use in the NICU environment or at home.
- Provide appropriate feedback that is also interpretable for parents and doctors or related people on whether the system’s components are functioning correctly.
- Look friendly, playful, familiar, and attractive to gain a feeling of trust from parents and clinicians.
- Be scalable to include more monitoring functions, such as wireless communication and local signal processing.
- Be made of easy-to-remove non-washable parts.

The placement of sensors on infants still needs to be further explored in the future, since it is difficult to know where the sensors could provide the most precise and relevant [62]. Data from variously-placed sensors need to be collected for diverse purposes, and it directly influences the exterior structure of system. Studies have indicated and implied that the placement of sensors take a profound influence over motion identification. In [17], Rihar et al. employed IMUs placed on the upper arm and forearm, in combination with a pressure mattress in order to measure the trunk position, rotation, and associated movements surface. Five IMUs were attached to the test subject, which were set inside the particularly-designed silicone bracelets. Singh et al. used accelerometers placed around the wrists and ankles of infant to predict cerebral palsy [19]. 

**Figure 3:**
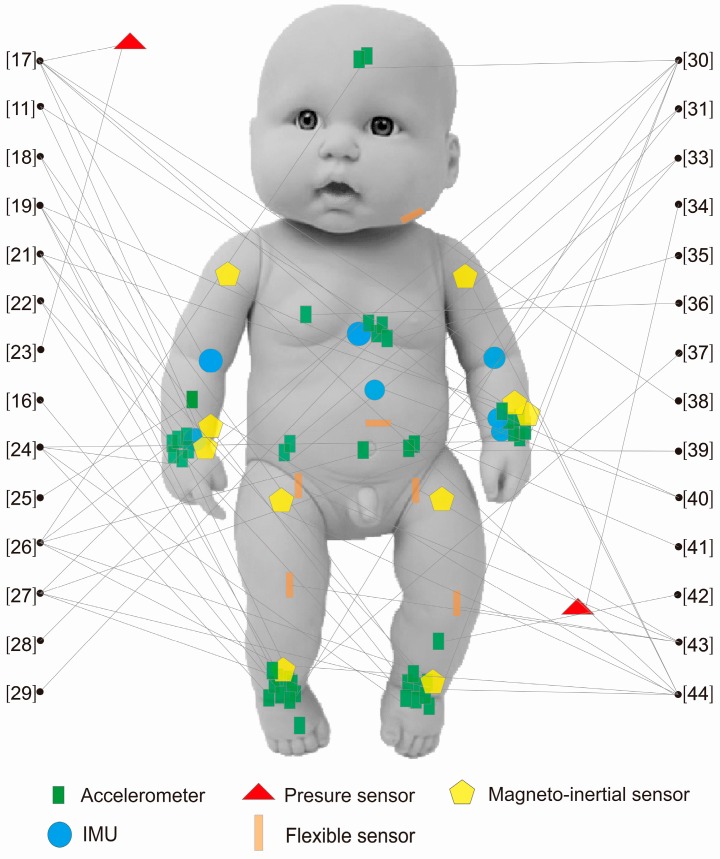
Infographic of sensor placements.

In Figure 3, from 2010–2016, most studies on infant movement choose to put the sensors at the position of wrist or ankle.

Furthermore, user acceptance is an important issue to consider during the system design. Affecting factors include infant comfort, the nature and level of user involvement in the development and implementation of the system, direct and indirect impacts of the new system on work practices, data reliability, battery life, etc.

As can be seen above, none of the 30 articles mentioned seizure detection with wearable systems, which is a promising area worthy of further study. Therefore, a more baby-friendly seizure detection system is in demand to be invented, developed, and verified by clinical trials.

## 5. Conclusions and Future Prospects

Among the technologies applied to infants’ movement monitoring, many researchers have chosen accelerometers and IMUs. They are lightweight, portable, and can monitor infants’ movements continuously. Meanwhile, sensor–based measurement of infant activities can provide quantitative assessment of infant movements. The studies that use accelerometers or IMUs to obtain infants’ motion patterns for diagnosis on neuro-motor development are very common. Existing literature on infants’ movement recognition using accelerometer data is widely varied in approaches, outcomes, and intentions. However, few studies use movement sensors to explore long-term monitoring of abnormal movement situation like seizure and emergency response for infants. In addition, approaches that combine various sensors providing more motion information have the potential to improve the accuracy of the motion measurements. 

Due to the miniaturization and lightweight of wearable motion sensors, they can be integrated into clothing or accessories from an ergonomic point of view [78]. Devices that attach to the wrist or ankle with a band seemed to be the most popular placement in those studies. To the best of our knowledge, up to now, there is no relevant study that examines the design of wearable sensor systems for infant movement monitoring from an interactive or ergonomic viewpoint. 

The current paper provides a systematic and detailed review of wearable sensor systems for monitoring infants’ movements. After the introduction of the significance of the wearable sensor system in movement monitoring for infants, the design fundamentals for wearable sensor-based movement detectors in infants, the appliance of movement monitoring in clinical practice, and the form and sensor placement in these systems, as explored. It can be foreseen that infant movement monitoring with wearable sensor systems will contribute significantly to the field of health care and neuron-motor development of infants. 

So far, much progress has been made in the area of wearable sensor system of infants’ movement monitoring and there are good prospects for future applications. New systems need to be developed and verified by more clinical trials before promotion to a wider population. For instance, the accuracy of the results obtained from the wearable motion sensors, the preferable choice to place the sensor for data collection in various applications, the reliability and comfort index of the system, and the aspects that could affect the results of the wearable sensors are expected to see improvement. Moreover, the application of wearable sensor-based movement monitoring in infants has not yet reached its full potential. The available literature does not show successful examples to detect infant seizure conditions based on the use of wearable motion sensors. Thus, there is a great development space of infant motion monitoring with wearable sensors for the detection of infant seizures. Another important trend and research direction is to create a “baby care system” to achieve feedback between clinicians and infants using wearable sensors. Through the “baby care system”, monitoring infants’ daily physical activities and understanding the real-time development status are within reach. Once these issues achieve any substantial progress, it will be a great attraction for both parents and clinicians for significant improved care of infants.

## Figures and Tables

**Table 1:** Literature search strategy (PubMed).

Infant	Infant OR Baby OR Neonatal OR Newborn
**AND**	
**Movement**	“Seizure activity” **OR** Convulsion **OR** “Motor behavior” **OR** Movement **OR** Position **OR** Motion **OR** Moving
**AND**	
**Monitoring**	Monitoring **OR** Feedback
**AND**	
**Wearable**	Wearable **OR** Mobile **OR** Ambulatory **OR** garment **OR** soft suit **OR** exosuit
**NEAR**	
**Sensor**	Accelerometer **OR** “Motion sensing” **OR** “Activity sensing” **OR** Gyroscope **OR** MEMS **OR** IMUs **OR** bend sensor **OR** flexible sensor

**Table 2:** Overview of the wearable sensor system to monitor infant movements.

Research Work	Year	Sensor	Placement	Form	Evaluation	Purpose
Rihar et al. [17]	2014	6 Wireless IMUs, 2 pressure mattresses	Trunk and arm	Silicone bracelets	Technical experiment (test baby doll) technical report user test	Infant motor pattern assessment
Taffoni et al. [11]	2012	2 Wired magneto-inertial sensor	Wrist	N/A	Technical experiment	Study motor skill at risk for autism spectrum (ASD)
Smith et al. [18]	2015	2 Inertial movement sensor(Opals, APDM) IMUs	Leg	Placed sensor on each leg using knee socks	Clinical test (*n* = 12)	Quantification of daily infant leg movements
Singh et al. [19]	2010	4 Custom Accelerometer (Eco)	Wrist and ankle	N/A	Clinical test (*n* = 10)	Predict CP
Saadatian et al. [20]	2011	1 Accelerometer	N/A	Wearable hardware gadget	Technical experiment	Baby care
Heinze et al. [21]	2010	4 Accelerometer	Extremities	N/A	Clinical test (*n* = 23)	Predict CP
Gima et al. [22]	2011	2 Accelerometer	Ankle	N/A	Clinical test (*n* = 8)	Infant motor pattern assessment
Boughorbel et al. [23]	2010	4 Pressure sensitive sensor	N/A	Mat	Technical experiment, Usability Evaluation (*n* = 1)	Infant care/SIDS
Lee, E. [16]	2015	1 Accelerometer	Ankle	Ankle band	Commercial product	Baby safety
Fan et al. [24]	2012	4 Accelerometer	Wrists and ankles	Clothes bands	Clinical validation (*n* = 10)	Infant motor pattern assessment/predict CP
Waldmeier et al. [25]	2013	1 Accelerometer	Hand	Fixed to the infant with a tape	Preclinical test, Usability Evaluation (*n* = 22)	Infant motor pattern assessment
Gravem et al. [26]	2012	5 Accelerometer	Ankle, wrists and forehead	Cloth bands	Clinical test (*n* = 10) Comparison Experiment	Infant motor pattern assessment/diagnosis CP
Abney et al. [27]	2014	4 Accelerometer	Wrist and ankle	N/A	Preclinical test, Usability Evaluation (*n* = 2)	Characterizations of infant behavioral development
Lin et al. [28]	2014	1 Accelerometer	Chest	Soft belt	Technical experiment	Prevent SIDS
Kaushik et al. [29]	2013	1 Accelerometer	Chest	Jacket	Technical experiment	Fall protection
Hayes et al. [30]	2011	5 Custom Accelerometer (Eco)	Ankle, wrists and forehead	Cloth bands	Preclinical test, Usability Evaluation (*n* = 10)	Infant motor pattern assessment/Predict CP
Jourand et al. [31]	2010	2 Accelerometer	Abdomen	N/A	Technical experiment	Monitor SIDS
López et al. [32]	2013	1 Accelerometer	N/A	Bear gadget	N/A	Prevent SIDS
Clercq et al. [33]	2010	2 Accelerometer	Abdomen	N/A	Technical experiment	Infant care/SIDS
Donati et al. [34]	2014	768 Pressure Sensor	N/A	Mat	Preclinical test, Usability Evaluation (*n* = 1)	Infant motor pattern
Fernandes [35]	2016	1 Accelerometer	Chest	Belt	Technical experiment	Monitor SIDS
Bouwstra, S et al. [36]	2011	1 Accelerometer	Right chest	Smark Jacket	Technical experiment	Motion artifacts reduction
Leier et al. [37]	2013	1 Accelerometer	Foot	Shoe	N/A	Baby safety
Farooq et al. [38]	2015	1 Jew Motion Sensor/Flexible sensor	Jaw	N/A	Clinical validation (*n* = 10)	Feeding Behavior
Huyen et al. [39]	2016	1 Accelerometer	Abdomen	Belt	Technical experiment	Baby safety
Rihar et al. [40]	2016	2IMU	Trunk and wrist	Bracelets and chest strap	Technical experiment	Infant motor development assessment/early intervention treatment
Koch et al. [41]	2016	Flexible 6 × 6 sensor	Abdomen	N/A	Technical experiment	Respiratory monitoring
Galland et al. [42]	2012	1 Accelerometer	Shin	N/A	Clinical validation (*n* = 33)	Sleep state monitoring
Rogers et al. [43]	2015	4 Joint angle sensors/Flexible sensor	Knees and hips	Sensing suit	Preclinical test, Usability Evaluation (*n* = 1)	Early intervention treatment
Karch et al. [44]	2012	Electromagnetic tracking system	upper and lower limb	N/A	Preclinical test (*n* = 75)	Predict CP

**Table 3:** Statistical representation of sensors selection for infant movement monitoring.

Category	Discussed by Papers
IMU	[17,18,40]
Accelerometer	[16,19,20,21,22,24,25,26,27,28,29,30,31,32,33,35,36,37,39,42]
Magneto-inertial	[11,44]
Pressure sensor	[17,23,34]
Flexible sensor	[38,41,43]

**Table 4:** Statistical representation of research purpose of infant movement monitoring.

Purpose	Discussed by Papers
Movement and motor pattern development	[17,18,21,22,23,25,26,27,30,38,40,42,43]
Cerebral palsy	[19,24,44]
Sleep safe/breathing rhythm/Sudden infant death syndrome (SIDS)/Prevent falls of infants/Autism spectrum disorders (ASD)	[11,16,20,28,29,31,32,33,34,35,37,39,41]

**Table 5:** Age-specific characteristics of normal GMs.

GM Type	Period of Presence in Weeks’ PMA	Description
Preterm GMs	From ± 28 weeks to 36–38 weeks	Great variation over time, more proximal than that in earlier days and are characterized by small to moderate amplitude and slow to moderate speed
Writhing GMs	From 36–38 weeks to 46–52 weeks	Seem to be somewhat slower and to show less participation of the pelvis and trunk.
Fidgety GMs	From 46–52 weeks to 54–58 weeks	Consists of a continuous flow of small and elegant movements, occur irregularly all over the body, head, trunk, and limbs participate to a similar extent

## References

[1] Gruskin A., Williams R.G., McCabe E.R.B., Stein F., Strickler J., Chesney R.W., Mulvey H.J., Simon J.L., Alden E.R. (2000). Final report of the FOPE II Pediatric Subspecialists of the Future Workgroup. Pediatrics.

[2] Pickler R.H., McGrath J.M., Reyna M.B.A., McCain N., Lewis M.M., Cone M.S., Wetzel P., Best A. (2010). A model of neurodevelopmental risk and protection for preterm infants. J. Perinat. Neonatal Nurs..

[3] Nzeh D.A., Erinle S.A., Saidu S.A., Pam S.D. (2004). Transfontanelle Ultra-Sonography: An Invaluable Tool in the Assessment of the Infant Brain. Trop. Dr..

[4] Pfefferbaum A., Mathalon D.H., Sullivan E.V., Rawles J.M., Zipursky R.B., Lim K.O. (1994). A quantitative magnetic resonance imaging study of changes in brain morphology from infancy to late adulthood. Arch. Neurol..

[5] Watanabe K., Hayakawa F., Okumura A. (1999). Neonatal EEG: A powerful tool in the assessment of brain damage in preterm infants. Brain Dev..

[6] Franceschini M.A., Thaker S., Themelis G., Krishnamoorthy K.K., Bortfeld H., Diamond S.G., Boas D.A., Arvin K., Grant P.E. (2007). Assessment of infant brain development with frequency-domain near-infrared spectroscopy. Pediatr. Res..

[7] Perlman J.M. (2004). Brain injury in the term infant. Semin. Perinatol..

[8] Rennie J.M. (1997). Neonatal seizures. Eur. J. Pediatr..

[9] Evans D., Levene M. (1998). Neonatal seizures. Arch. Dis. Child. Fetal Neonatal Ed..

[10] Einspieler C., Heinz F.R. (2005). Prechtl’s assessment of general movements: A diagnostic tool for the functional assessment of the young nervous system. Ment. Retard. Dev. Disabil. Res. Rev..

[11] Taffoni F., Focaroli V., Formica D., Gugliemelli E. Sensor-based technology in the study of motor skills in infants at risk for ASD. Proceedings of the IEEE International Conference on Biomedical Robotics and Biomechatronic.

[12] Chan M., Estève D., Fourniols J.-Y., Escriba C., Campo E. (2012). Smart wearable systems: Current status and future challenges. Artif. Intell. Med..

[13] Zhu Z., Liu T., Li G., Li T., Inoue Y. (2015). Wearable Sensor Systems for Infants. Sensors.

[14] Marcroft C. (2015). Movement Recognition Technology as a Method of Assessing Spontaneous General Movements in High Risk Infants. Front. Neurol..

[15] Wikipedia. https://en.wikipedia.org/wiki/Wearable_technologyI.

[16] Lee E. (2015). Baby by the numbers [Resources Tools]. IEEE. Spectr..

[17] Rihar A., Mihelj M., Pasic J., Munih M. (2014). Infant trunk posture and arm movement assessment using pressure mattress; inertial and magnetic measurement units (IMUs). J. Neuroeng. Rehabil..

[18] Smith B.A., Trujillo-Priego I.A. (2015). Daily Quantity of Infant Leg Movement. Wearable Sensor Algorithm and Relationship to Walking Onset. Sensors.

[19] Singh M., Patterson D.J. Involuntary gesture recognition for predicting cerebral palsy in high-risk infants. Proceedings of the International Symposium on Wearable Computers (ISWC).

[20] Saadatian E., Iyer S.P., Lihui C. Low cost infant monitoring and communication system. Proceedings of the 2011 IEEE Colloquium on Humanities, Science and Engineering (CHUSER).

[21] Heinze F., Hesels K., Breitbach-Faller N., Schmitz-Rode T., Disselhorst-Klug C. (2010). Movement analysis by accelerometry of newborns and infants for the early detection of movement disorders due to infantile cerebral palsy. Med. Biol. Eng. Comput..

[22] Gima H., Ohgi S., Morita S., Karasuno H., Fujiwara T., Abe K. (2011). A dynamical system analysis of the development of spontaneous lower extremity movements in newborn and young infants. J. Physiol. Anthropol..

[23] Boughorbel S., Bruekers F., Breebaart J. Baby-Posture Classification from Pressure-Sensor Data. Proceedings of the 2010 20th International Conference on Pattern Recognition (ICPR).

[24] Fan M., Gravem D., Cooper D.M., Patterson D.J. Augmenting Gesture Recognition with Erlang-cox Models to Identify Neurological Disorders in Premature Babies. Proceedings of the 2012 ACM Conference on Ubiquitous Computing.

[25] Waldmeier S., Grunt S., Delgado-Eckert E., Latzin P., Steinlin M., Fuhrer K., Frey U. (2013). Correlation properties of spontaneous motor activity in healthy infants: A new computer-assisted method to evaluate neurological maturation. Exp. Brain Res..

[26] Gravem D., Singh M., Chen C., Rich J., Vaughan J., Goldberg K., Waffarn F., Chou P., Cooper D., Reinkensmeyer D. (2012). Assessment of Infant Movement with a Compact Wireless Accelerometer System. J. Med. Devices.

[27] Abney D.H., Warlaumont A.S., Hanussman A., Ross J.M., Wallot S. (2014). Using nonlinear methods to quantify changes in infant limb movements and vocalizations. Front. Psychol..

[28] Lin W., Brittelli J., Lehmann C. Wireless Infant Monitoring Device for the prevention of sudden infant death syndrome. Proceedings of the 2014 11th International Conference & Expo on IEEE Emerging Technologies for a Smarter World (CEWIT).

[29] Kaushik A., Singh R. (2013). Infant Monitoring and Fall Avoidance System using Tri-Axial Accelerometer and ARM7 Microcontroller. Int. J. Comput. Appl..

[30] Hayes G.R., Patterson D.J., Singh M., Gravem D., Rich J. (2011). Supporting the transition from hospital to home for premature infants using integrated mobile computing and sensor support. Pers. Ubiquitous Comput..

[31] Jourand P., De Clercq H., Puers R. (2010). Robust monitoring of vital signs integrated in textile. Sens. Actuators A Phys..

[32] López G., López M., Guerrero L.A., Nugent C., Coronato A., Bravo J. (2013). An Augmented Object Prototype for Helping to Prevent the Sudden Infant Death Syndrome. Proceedings of the International Workshop on Ambient Assisted Living.

[33] De Clercq H., Jourand P., Puers R. Textile Integrated Monitoring System for Breathing Rhythm of Infants. Proceedings of the XII Mediterranean Conference on Medical and Biological Engineering and Computing.

[34] Donati M., Cecchi F., Bonaccorso F., Branciforte M., Dario P., Vitiello N. (2013). A modular sensorized mat for monitoring infant posture. Sensors.

[35] Fernandes D., Cabral J., Rocha A.M. (2016). A smart wearable system for sudden infant death syndrome monitoring. Proceedings of the 2016 IEEE International Conference on Industrial Technology (ICIT).

[36] Bouwstra S., Chen W., Oetomo S.B., Feijs L.M.G., Cluitmans P.J.M. (2011). Designing for reliable textile neonatal ECG monitoring using multi-sensor recordings. Proceedings of the 2011 Annual International Conference of the IEEE Engineering in Medicine and Biology Society.

[37] Leier M., Jervan G. (2013). Sleep apnea pre-screening on neonates and children with shoe integrated sensors. Proceedings of the NORCHIP.

[38] Farooq M., Chandler-Laney P., Hernandez-Reif M., Sazonov E. (2015). Monitoring of infant feeding behavior using a jaw motion sensor. J. Healthc. Eng..

[39] Vu H., Eftestøl T., Engan K., Eilevstjønn J., Yarrot L.B., Linde J.E., Ersdal H. (2016). Detection of Activities during Newborn Resuscitation Based on Short-Time Energy of Acceleration Signal. Proceedings of the International Conference on Image and Signal Processing.

[40] Rihar A., Sgandurra G., Beani E., Cecchi F., Pašič J., Cioni G., Dario P., Mihelj M., Munih M. (2016). CareToy: Stimulation and Assessment of Preterm Infant’s Activity Using a Novel Sensorized System. Ann. Biomed. Eng..

[41] Koch E., Dietzel A. (2016). Skin attachable flexible sensor array for respiratory monitoring. Sens. Actuators A Phys..

[42] Galland B.C., Kennedy G.J., Mitchell E.A., Taylor B.J. (2012). Algorithms for using an activity-based accelerometer for identification of infant sleep–wake states during nap studies. Sleep Med..

[43] Rogers E., Polygerinos P., Walsh C., Goldfield E. (2015). Smart and Connected Actuated Mobile and Sensing Suit to Encourage Motion in Developmentally Delayed Infants. J. Med. Devices.

[44] Karch D., Kang K.S., Wochner K., Philippi H., Hadders-Algra M., Pietz J., Dickhaus H. (2012). Kinematic assessment of stereotypy in spontaneous movements in infants. Gait Posture.

[45] Yazdi N., Ayazi F., Najafi K. (1998). Micromachined inertial sensors. Proc. IEEE.

[46] Seel T., Raisch J., Schauer T. (2014). IMU-based joint angle measurement for gait analysis. Sensors.

[47] Yang S., Li Q. (2012). Inertial sensor-based methods in walking speed estimation: A systematic review. Sensors.

[48] Asokanthan S.F., Wang T. (2009). Instabilities in a MEMS gyroscope subjected to angular rate fluctuations. J. Vib. Control.

[49] Casey P.H., Bradley R.H., Whiteside-Mansell L., Barrett K., Gossett J.M., Simpson P.M. (2009). Effect of early intervention on 8-year growth status of low-birth-weight preterm infants. Arch. Pediatr. Adolesc. Med..

[50] Cameron E.C., Maehle V., Reid J. (2005). The effects of an early physical therapy intervention for very preterm; very low birth weight infants: A randomized controlled clinical trial. Pediatr. Phys. Ther..

[51] Spittle A.J., Doyle L.W., Boyd R.N. (2008). A systematic review of the clinimetric properties of neuromotor assessments for preterm infants during the first year of life. Dev. Med. Child Neurol..

[52] Cioni G., Prechtl H.F., Ferrari F., Paolicelli P.B., Einspieler C., Roversi M.F. (1997). Which better predicts later outcome in full-term infants: Quality of general movements or neurological examination. Early Hum. Dev..

[53] Ferrari F., Cioni G., Prechtl H.F.R. (1990). Qualitative changes of general movements in preterm infants with brain lesions. Early Hum. Dev..

[54] Hadders-Algra M. (2004). General movements: A window for early identification of children at high risk for developmental disorders. J. Pediatr..

[55] Bos A.F. (1993). Differential effects of brain lesions and systemic disease on the quality of general movements: A preliminary report. Early Hum. Dev..

[56] (2015). WIKI. https://en.wikipedia.org/wiki/Cerebral_palsy.

[57] Trapp B.E. (2010). National Institute of Neurological Disorders and Stroke. J. Consum. Health Internet.

[58] Ferrari F., Cioni G., Einspieler C., Rocersi M.F., Bos A.F., Paolicelli P.B., Ranzi A., Oversi H.F.R. (2002). CRamped synchronized general movements in preterm infants as an early marker for cerebral palsy. Arch. Pediatr. Adolesc. Med..

[59] Cui X. (2014). The Firmware Development of a Portable Inertial Measurement Unit (IMU). https://opus.lib.uts.edu.au/handle/10453/35940.

[60] Nordli D.R., Bazil C.W., Scheuer M.L., Pedley T.A. (1997). Recognition and Classification of Seizures in Infants. Epilepsia.

[61] Lockman J., Fisher R.S., Olson D.M. (2011). Detection of seizure-like movements using a wrist accelerometer. Epilepsy Behav..

[62] Kamalizonouzi B. (2012). Optimal Inertial Sensor Placement and Motion Detection for Epileptic Seizure Patient Monitoring. Master’s Thesis.

[63] Van de Vel A., Cuppens K., Bonroy B., Milosevic M., Van Huffel S., Vanrumste B., Lagae L., Ceulemans B. (2013). Long-term home monitoring of hypermotor seizures by patient-worn accelerometers. Epilepsy Behav..

[64] Decaigny A.S., Cuppens K., Lagae L., Ceulemans B., Van Huffel S., Vanrumste B. Accelerometers used for the detection of nocturnal frontal lobe seizures in pediatric patients. Proceedings of the European Conference on the Use of Modern Information and Communication (ECUMICT).

[65] Lundqvist-Persson C., Lau G., Nordin P., Bona E., Sabel K.G. (2012). Preterm infants’ early developmental status is associated with later developmental outcome. Acta Paediatr..

[66] Mayo N.E. (1991). The Effect of Physical Therapy for Children with Motor Delay and Cerebral Palsy. Am. J. Phys. Med. Rehabil..

[67] Al-Dasoqi N., Mason A., Shaw A., Al-Shamma’a A.I. Preventing cot death for infants in day care. Proceedings of the 2010 IEEE Sensors Applications Symposium (SAS).

[68] Khan I.M., Jabeur N., Khan M.Z., Mokhtar H. An overview of the impact of wireless sensor networks in medical health care. Proceedings of the 1st International Conference on Computing and Information Technology (ICCT).

[69] De Jonge G.A., Engelberts A.C., Koomen-Liefting A.J., Kostense P.J. (1989). Cot death and prone sleeping position in The Netherlands. Br. Med. J..

[70] Dwyer T., Ponsonby A.-L. (2009). Sudden Infant Death Syndrome and Prone Sleeping Position. Ann. Epidemiol..

[71] Mitchell E.A., Engelberts A.C., Bettelheim K.A., Smith H., Goldwater P.N., Morris J.A., Murrell T.G.C., Sweet C., Weaver S.A. (1991). Sleeping position and cot deaths. Lancet.

[72] (2010). American SIDS Institute Website. http://www.sids.org.

[73] Sudharsanan S., Karthikeyan B. (2013). The design of a real-time accelerometer-based baby sleeping position monitoring and correction system. Int. J. Biomed. Eng. Technol..

[74] Hung P.D., Bonnet S., Guillemaud R., Castelli E., Yen P.T.N. Estimation of respiratory waveform using an accelerometer. Proceedings of the 2008 5th IEEE International Symposium on Biomedical Imaging: From Nano to Macro.

[75] Clifford G.D., Long W.J., Moody G.B., Szolovits P. (2009). Robust parameter extraction for decision support using multimodal intensive care data. Philos. Trans. R. Soc. Lond. A Math. Phys. Eng. Sci..

[76] Bourbakis N.G. (2010). A Survey on Wearable Sensor-Based Systems for Health Monitoring and Prognosis. IEEE Trans. Syst. Man Cybern. C.

[77] Bouwstra S., Chen W., Feijs L., Oetomo S.B. Smart Jacket Design for Neonatal Monitoring with Wearable Sensors. Proceedings of the Sixth International Workshop on Wearable and Implantable Body Sensor Networks.

[78] Yang C.C., Hsu Y.L. (2010). A review of accelerometry-based wearable motion detectors for physical activity monitoring. Sensors.

